# Why water security matters to cities under extreme heat in the Global North

**DOI:** 10.1038/s42949-025-00317-6

**Published:** 2026-01-20

**Authors:** Hug March, Katie Meehan, Elena Domene, Mar Satorras, David Saurí

**Affiliations:** 1 Estudis d’Economia i Empresa, Universitat Oberta de Catalunya, Barcelona, Spain; 2 Department of Geography, King’s College London, London, UK; 3 Institut Metròpoli, Universitat Autònoma de Barcelona, Bellaterra, Spain; 4 Departament de Geografia, Universitat Autònoma de Barcelona, Bellaterra, Spain

**Keywords:** Environmental studies, Social policy

## Abstract

Household water security — safe, affordable, reliable, and acceptable water for a thriving life — is a key avenue for adapting to extreme and chronic heat, particularly in cities. We argue that household water security is far from universal in the urban Global North, resulting in uneven capacities and strategies to adapt to heat, outside and within the home. We synthesize key insights to advance water security as a central plank of urban heat justice.

## Introduction

The summer of 2025 brought Europe to a boiling point. Record-breaking heatwaves swept the continent starting in mid-June, with temperatures soaring above 46 °C in parts of Spain and Portugal^1^. In Barcelona, nights were torrid, with minimum temperatures above 25 °C for 10 days^2^, making restful sleep nearly impossible. Against this backdrop, cities such as Paris and Barcelona are bracing for a future where daily highs of 50 °C could be reached^3,4^. Conditions of extreme heat are already straining public health systems^5^, leading to spikes in hospitalizations^6^, especially in older adults^7^, and increased mortality^8^. It is estimated that the 10 days of extreme heat in late June and early July 2025 in Europe resulted in ~2300 excess deaths across 12 major European cities, with 65% of the deaths attributed to the effects of climate change^9^. Furthermore, heatwaves interact with the Urban Heat Island effect in ways that may exacerbate heat stress in cities compared to suburban and rural areas^10^.

Media coverage of the summer 2025 heatwave in urban Europe included photographs of residents, tourists, and workers crowded around public fountains, dipping their heads and bodies into the water in a bid for relief^11^. Indeed, this imagery reminds us of the powerful yet poorly understood interconnections among urban heat, climate change adaptation, and water security—raising questions of how urban dwellers living in poverty, without reliable, affordable, or secure access to water, can adapt or survive in the “new normal” of heat^12^.

In a recent article, Anguelovski et al.^13^ call for a justice-oriented approach to urban heat governance in the USA and Europe, arguing that uneven exposure to heat in cities, fueled by a legacy of exclusionary socio-spatial policies (class-based and racialized), is reinforced by the very responses to heat. Their framing of “heat racism” and “heat gentrification” offers a lens to understand the socio-spatial dynamics of vulnerability to extreme heat and the active production of vulnerability through gentrification and urban redevelopment. However, while their analysis rightly emphasizes the intersection of housing, green infrastructure, and access to cooling spaces, it only briefly touches on water—in the context of climate shelters in Barcelona—and water remains completely delinked from the housing question. Simply put, the framework of “heat justice” by Anguelovski et al.^13^ *takes for granted the existence of secure water* in the USA and Europe—an assumption that is contested by a growing body of evidence^14–18^. In advancing the justice-oriented heat agenda, *we argue that household water security*—affordable, reliable, safe, and acceptable water^19^—*is a prime avenue for heat adaptation*, *both within and outside of the ‘home’*, *and represents a critical, yet underexplored, dimension of heat justice in the urban Global North*. To help close this gap, we offer two key insights from the emerging literature on household water insecurity to advance the broader research agenda on heat justice.

## Water is a tool for household adaptation to heat

The baseline of heat is shifting. Extreme hydroclimatic events—particularly heatwaves and droughts—are becoming more frequent, intense, and *chronic*, meaning they are likely to persist^20^. For instance, the heatwave intensity in the Metropolitan Area of Barcelona is projected to increase by 2.5 °C by 2050 and 4.2 °C by 2100^21^. While mitigation remains essential, adaptation is urgent, especially in regions such as the Mediterranean or the US Southwest, where climate change amplifies heat and extends the seasonality of “heat extremes”^22^.

Extreme heat is a killer^23^ and encompasses both acute events (heatwaves) and chronic exposure, with well-documented impacts on comfort, wellbeing, physical activity, sleep quality, mental health, and exacerbating chronic diseases^24–27^. These effects are not evenly distributed: age, gender, health status, socioeconomic status, housing conditions, and access to energy and water overlap and compound to shape vulnerability^5,28–31^. Indeed, the emerging field of critical heat studies points to the need to develop a research agenda on the social production of heat and thermal (in)security^32^.

In this context, water emerges as a vital tool for human cooling and survival at the household level, particularly for people who cannot afford energy-fueled cooling technologies or lack access to “climate shelters”^12,17,33^. Even in studies focused on heat adaptation in low-income households, such as Palinkas et al.^34^ for California, US, or Osberghaus and Abeling^35^ for Germany, or community-based heat adaptation interventions^36^, water is rarely discussed as a direct cooling resource, only for direct hydration (Erens et al.^37^ for the UK or Hayden et al.^38^ for the US). Still, we found empirical evidence (primarily through surveys) from the Global North identifying water-based cooling behaviors other than hydration, such as cold showers, wetting the body, or using public water sources (Table 1).

**Table 1:** Surveys identifying water-based cooling behaviors

Reference	Region	Water-related strategies addressed
Zander et al.^27^	Australia	Water consumption
Palinkas et al.^34^	LA, USA (low-income)	Stay hydrated
		Keep cool (includes taking showers)
Hayden et al.^38^	Texas, US	Drink water
		Take cool showers
Nitschke et al.^56^	Australia (older people)	Extra cool showers
		Use a wet cloth on the neck and face
		Drink more fluid
Hatvani-Kovacs et al.^57^	Australia	Drink plenty of water
		Cooling showers
		Go to a swimming pool
		Go to the beach or the hills
Beckmann et al.^58^	Germany	Taking showers at night
Satorras et al.^48^	Spain	Drink water
		Take showers
		Use swimming pools
		Use public fountains
Lao et al.^59^	China	Outdoor cooling practices
Lopes et al.^60^	Portugal	Increase fluid consumption
		Cool the body, for instance, by taking a shower
Akompab et al.^61^	Australia	Drink plenty of water to stay hydrated
Khare et al.^62^	UK	Cold drinks
Kunz-Plapp et al.^63^	Germany	Drink
		Cool body (*though not explicitly with water*)
Laranjeira et al.^64^	Germany	Adaptation of personal habits (*though not detailed*)
Zander et al.^65^	Australia	Change eating/water intake habits
		Showers only appear at the qualitative analysis: “*I have usually relied on fans and open windows for cooling and had cold showers/gone for a swim to beat the heat. However, over the last couple of years, due to the higher-than-average temperatures, I’ve had to run air-conditioning more often.”*

For households experiencing systemic cooling poverty, a deprivation rooted in inadequate infrastructure and resources, water becomes a primary, often lower-cost means of coping with heat^39^. As illustrated in the case of Singapore, water replaces energy in lower-income households without AC as the critical vector to find relief from heat^40^. These findings align with those of Qin et al.^41^ in the Chinese context, underscoring the critical importance of water as a widely used heat coping strategy in lower-income households. For instance, night showers are a strategy explicitly recognized by the European Insomnia Network to facilitate inducing sleep during heatwaves: “Before going to bed, with high ambient temperatures due to heatwaves, a cool or lukewarm shower (not cold) can help to induce sleep and reduce stress due to heat. Alternatively, a lukewarm foot bath can be effective”^25^. Water, simply put, is the original “air conditioner” of the urban poor, and water insecurity may jeopardize such an essential function. While secure water is widely recognized as essential for hydration and hygiene, its crucial role as a direct household cooling strategy during extreme heat remains underexplored in heat justice scholarship, and more widely in critical heat studies and debates around secure water. This gap is particularly relevant given the potential of water-based practices—such as cold showers, evaporative cooling, and surface wetting—to mitigate heat stress and improve comfort and sleep quality in vulnerable populations.

## Household water security is not a given

Without secure access to water, adaptive actions like staying hydrated or cooling the body become inaccessible, exacerbating health risks, deepening inequality, and threatening lives. Even in so-called “advanced” capitalist economies like the USA, household water security is not universal^15^ and is unevenly experienced across lines of class, race, and social differences^14,42^. Issues of household affordability, access, reliability, safety, and trust in water services are serious concerns in low-income and marginalized communities^43,44^.

Examples from two different regions serve to briefly underscore the high stakes of “heat justice” for households experiencing insecurity and water poverty. In several major US cities, there are now more households without access to running water than there were two decades ago—a stunning reversal of progress^16^. In Portland (OR), the number of households without running water increased by 56.3% between 2000 and 2021. Among Black Portlanders (11% of all Portlanders without running water), the proportionate increase nearly quadrupled (359%)^16^. Meanwhile, the Portland City Council recently announced a 6.34% rate increase for water and sewer tariffs, projecting annual household bills of $2400 per household by 2030^45^. These charges sit on top of mounting costs—for utility bills, as well as food, healthcare, childcare, education, transportation, internet, and other essential goods and services—that, together, function to “squeeze” the ability of low-income households to survive. What happens during a heatwave if water bills are missed and households are disconnected—a common practice in US cities^46^? In 2021, a deadly “heat dome” (with temperatures over 37 °C) killed 96 Portlanders^47^. A recent study found that 58% of the state’s most vulnerable population, including individuals living in mobile homes and farmworker housing, lacked sufficient cooling equipment in their homes, leaving water as the primary cooling option^47^.

In Southern Europe, where urban areas are experiencing recurrent and intensifying heat extremes, water-related practices such as showering, wetting the body, and hydration are part of the strategies employed, especially by low-income households, to cope with the heat. For instance, in the metropolis of Barcelona, drinking water and showering were mentioned among the top five practices adopted by households living in socially vulnerable areas experiencing extreme heat. Outside of homes, water was also embedded in heat coping practices such as using public swimming pools (a practice adopted by 37.6% of the respondents) or using public fountains to drink water or to cool off by wetting their face or head (adopted by 29.6%)^48^. In the Heat Watchers in Action project, children reported drinking more water and showering more often among the most recurring practices to cope with heat. More frequent and cooler showers, as well as public fountains, were among the top ideas proposed for future adaptation^49^. Along these lines, Saurí et al.^12^ report that taking baths or showers was a key cooling tactic in the hot Barcelona summers—a strategy that put lower-income households at risk due to financial pressures:

“As one interviewee insisted, *we must take short showers, especially my daughter, who’s the one spending more time*, while another complained that his son did not care and that *he would sleep in the shower*. The objective was therefore to shorten shower times and to eliminate baths: *In summer my daughter does not take baths anymore. It is especially important to educate her*. Self-imposing behavior became sometimes extremely strict with little room for comfort”^12^.

Looking forward, for cities in drought-prone areas, such as the US Southwest or Mediterranean, the cost of living for low-income households might be exacerbated by price hikes to “securitize” new water flows from unconventional sources such as desalination or, more recently, indirect potable reuse to make water systems “climate-proof”^50^. The substantial sunk and operational costs, particularly those linked to energy use, associated with these infrastructural upgrades^51^, which could also intersect with changing political economies of water supply (e.g., privatization and /or financialization), may paradoxically exacerbate water insecurity for the low-income populations by placing additional pressure on the affordability of water^52^ and hence putting extra pressure on water access for heat adaptation.

## Putting water on the heat justice agenda

In this contribution, we synthesized two major contributions from the emerging literature to advance the broader research agenda on heat justice. First, water is an underrecognized but vital tool for household adaptation to extreme heat in cities, especially for low-income and marginalized people. Second, household water security is far from universal in the Global North, meaning that the geography of survival in the “long heat” is fundamentally uneven. Moving forward, a truly transformative heat justice agenda *must* encompass matters of water security. Anguelovski et al.^13^ correctly focus on justice in urban heat governance in the Global North context. We extend this call by emphasizing that household water security is a foundational, yet overlooked, pillar of heat justice. In advancing research and practice, we urge studies that explicitly interrogate the mechanisms of power that *produce* conditions of heat inequality. An expanded research agenda for heat justice might include the following questions:

- Who is most affected by heat and water insecurities in the Global North, and why?
- How do existing urban governance structures reproduce or mitigate heat and water injustices?
- What is the relationship between household water insecurity and heat adaptation?
- What methods, data, and conceptual frameworks capture the lived experience of heat and water insecurity in high-income contexts^19^?

We spotlight the global work of the HWISE network, which developed the first validated scale for measuring household water insecurity across over 30 low- and middle-income countries. Recently, HWISE has expanded its focus and metrics to include high-income countries, such as the USA, to close the knowledge gap^53^. As a next step, a novel heat module for the HWISE survey may offer essential insights into how water shapes adaptive capacity in the face of rising temperatures. This would also pave the way for explicitly interrogating justice-oriented questions around power, inequality, and recognition in systemic cooling poverty and bring about a nexus dimension (water-heat) to the equation.

Extreme heat, including chronic heat^54^, is here to stay. Water is essential to guarantee heat adaptation in cities, both in public and private spaces. In public spaces, fountains for drinking water purposes, swimming pools to cool the body, and, more generally, green social infrastructures serving as climate shelters and addressing intersecting vulnerabilities are well-known options^55^. However, the household dimension of the nexus between water and heat in the Global North, where household water security is often taken for granted as universal, becomes critical in times of climate emergency, intersecting with systemic cooling poverty, which remains relatively unexplored^39^. Without secure, affordable, safe, and reliable water, the ability to adapt to chronic and extreme heat is fundamentally compromised. Also, it deepens existing social and spatial inequalities, particularly among vulnerable urban populations.

## Data Availability

No datasets were generated or analyzed during the current study.
